# Chilblains With Tumid Lupus Features in a Patient With Sjögren’s Syndrome: A Case Report

**DOI:** 10.7759/cureus.82439

**Published:** 2025-04-17

**Authors:** Thanda Aung, Mia Celestin

**Affiliations:** 1 Rheumatology, University of California Los Angeles, David Geffen School of Medicine, Los Angeles, USA

**Keywords:** belimumab, chilblains, perniosis, sjögren’s syndrome, tumid lupus

## Abstract

Sjögren's syndrome is an autoimmune disorder characterized by lymphocytic infiltration of exocrine glands, primarily affecting the lacrimal and salivary glands, resulting in dry eyes and mouth. We report a case of a 22-year-old female with Sjögren's syndrome (diagnosed at age 14), who developed chilblain-like lesions with histopathological features of tumid lupus. The patient presented with low-grade fever and painful erythematous papules on the fingers, toes, ears, face, and trunk. Serological workup revealed multiple autoantibodies (anti-SSA (Sjögren's-syndrome-related antigen A), anti-SSB (Sjögren's-syndrome-related antigen B), anti-centromere, anti-U1RNP (anti-U1 ribonucleoprotein), ANA (antinuclear antibody), and rheumatoid factor). Skin biopsy demonstrated interface dermatitis with lymphoplasmacytic infiltration and increased dermal mucin. After an inadequate response to conventional therapy (hydroxychloroquine, methotrexate, prednisone, and topical treatments), the patient showed significant improvement with belimumab. This case highlights the overlap between Sjögren's syndrome and lupus spectrum disorders. It demonstrates the potential efficacy of B-cell-targeted therapy in managing refractory cutaneous manifestations of autoimmune overlap syndromes.

## Introduction

Sjögren's syndrome is a chronic autoimmune inflammatory disorder characterized by lymphocytic infiltration of exocrine glands, primarily affecting salivary and lacrimal glands [1]. While glandular manifestations are the hallmark of the disease, extra-glandular involvement, including cutaneous manifestations, occurs in approximately 55% of patients [2,3]. Various dermatological presentations have been reported in Sjögren's syndrome, including xerosis, annular erythema, vasculitis, and less commonly, chilblains [4,5]. 

Chilblains (perniosis) present as inflammatory, erythematous to violaceous papules or nodules, typically affecting acral sites in response to cold exposure. Its pathophysiology involves abnormal vascular responses to cold, resulting in vasospasm followed by reactive vasodilation, with subsequent inflammation [6,7]. While primary chilblains often occur in otherwise healthy individuals, secondary chilblains can be associated with various autoimmune conditions, particularly lupus erythematosus (chilblain lupus) [7,8].

Tumid lupus erythematosus represents a distinct subtype of cutaneous lupus characterized by photosensitive, edematous, indurated plaques without epidermal involvement. Histopathologically, it features prominent dermal mucin deposition with perivascular and periadnexal lymphocytic infiltration [8,9]. The overlap between tumid lupus and chilblains in patients with Sjögren's syndrome has been rarely reported and presents diagnostic and therapeutic challenges. 

We present a case of chilblain-like lesions with histopathological features of tumid lupus in a young woman with Sjögren's syndrome, who demonstrated significant improvement with belimumab therapy after inadequate response to conventional treatments.

## Case presentation

**Figure 1:**
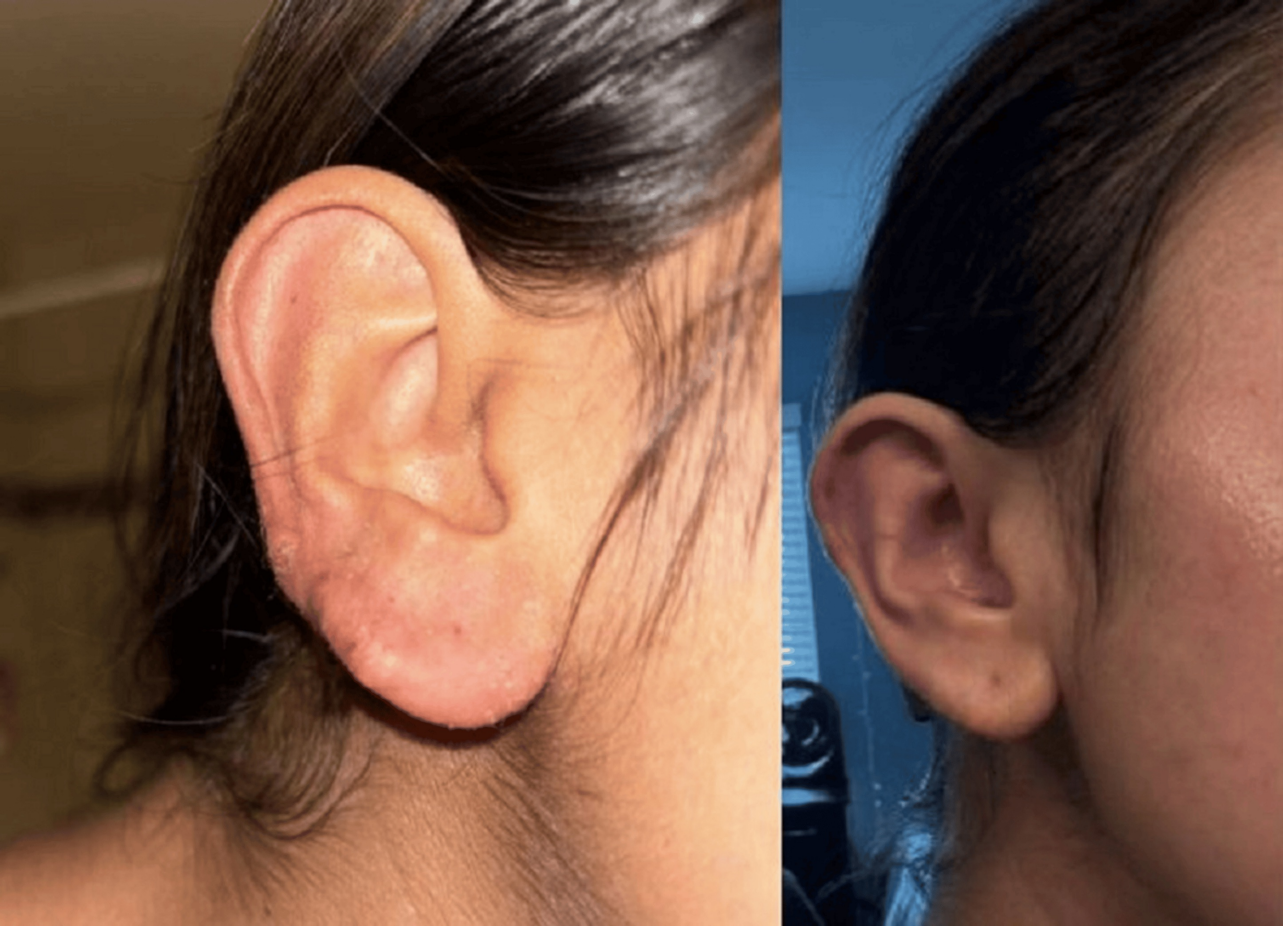
Lesion on the right ear before treatment with belimumab (left side), vs. resolution of lesions after treatment with belimumab (right side)

**Figure 2:**
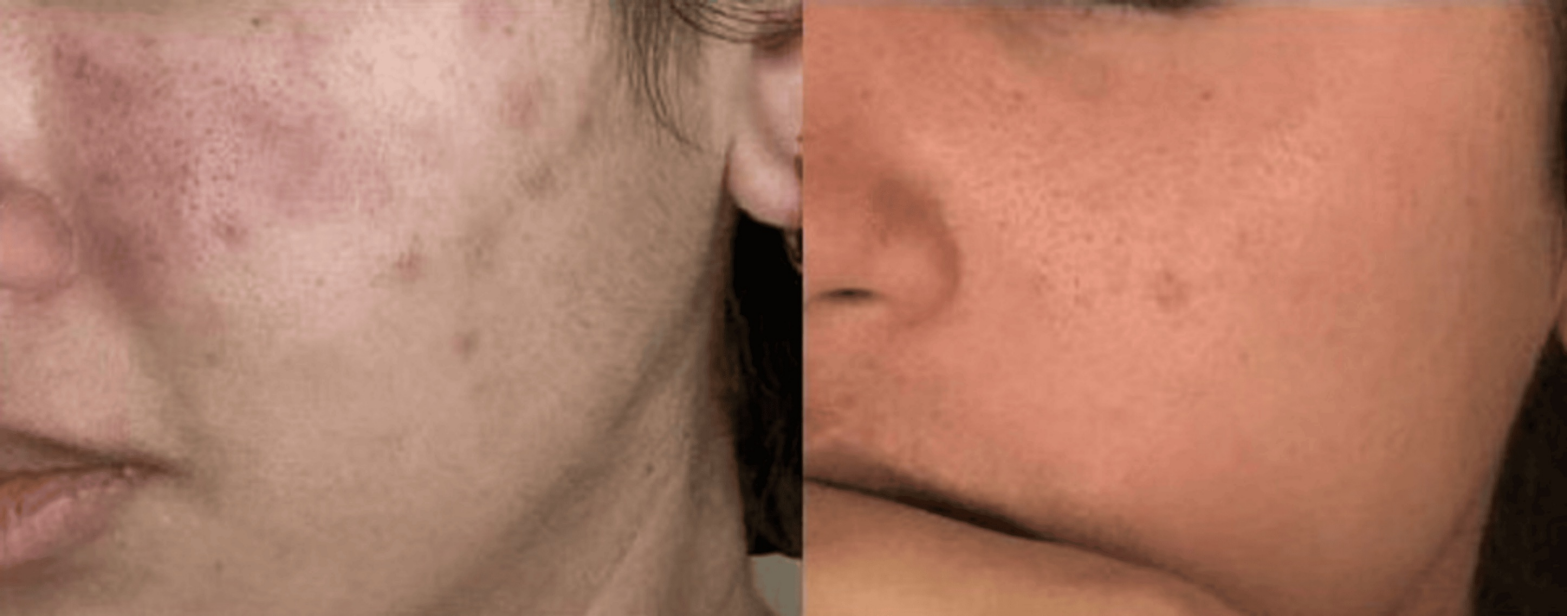
Lesion on the left cheek before treatment with belimumab (left side), vs. resolution of lesions after treatment with belimumab (right side)

**Figure 3:**
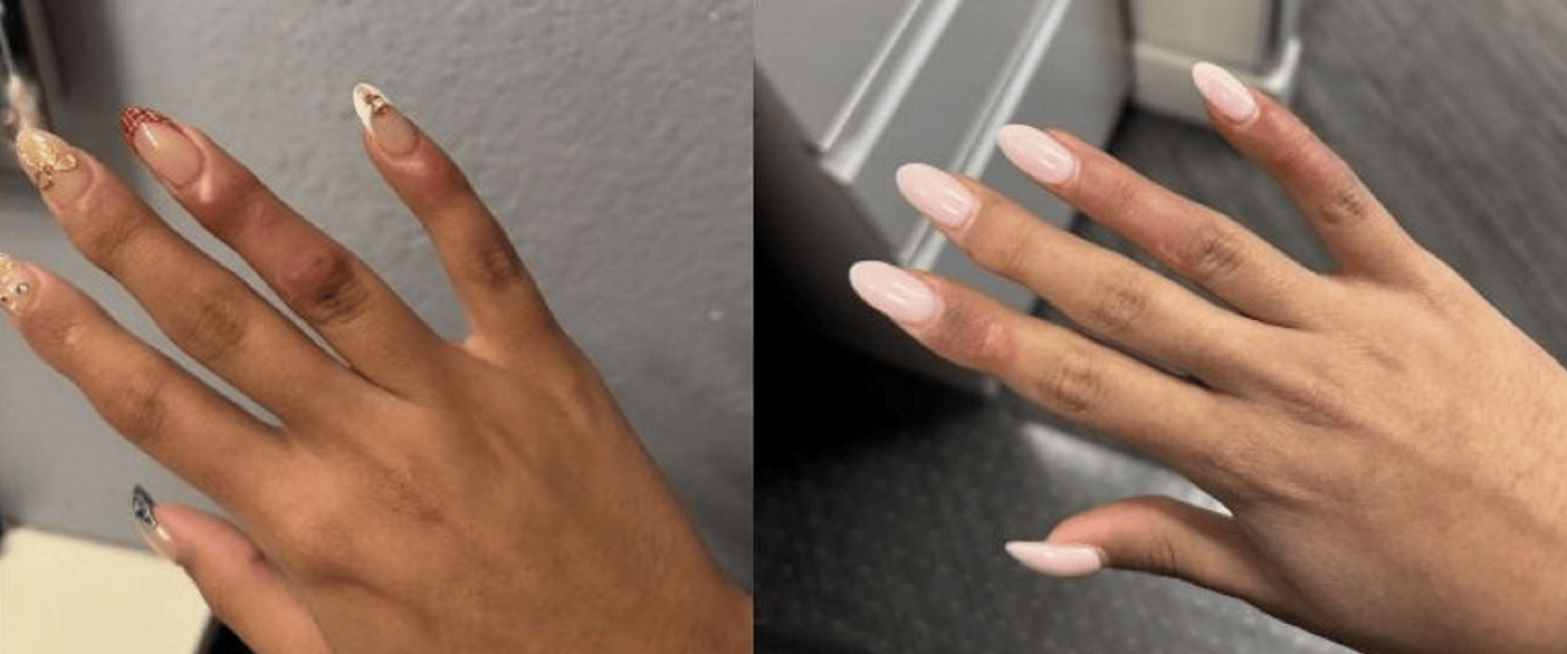
Lesion on the right hand before treatment with belimumab (left side), vs. resolution of lesions after treatment with belimumab (right side)

A 22-year-old female with Sjögren's syndrome, diagnosed at age 14, presented with low-grade fever and painful erythematous papules affecting the fingers, toes, and ears, as well as similar lesions on the face and trunk. Her medical history was significant for Raynaud's phenomenon. Physical examination revealed well-demarcated, erythematous to violaceous papules and plaques on the distal aspects of the fingers and toes, with similar lesions on the helices of both ears (Figures 1-3). Additional examination showed scattered erythematous, edematous plaques on the malar region of the face, and the upper back and chest. The lesions were tender to palpation, and the acral lesions became more prominent with cold exposure. 

Laboratory investigations revealed a normal complete blood count, as well as normal renal and liver function tests. Serological studies showed positive anti-SSA (Sjögren's-syndrome-related antigen A) (Ro) and anti-SSB (Sjögren's-syndrome-related antigen B) (La) antibodies, consistent with her established diagnosis of Sjögren's syndrome. Additional serologies revealed positive anti-centromere antibodies, anti-U1 SNRNP (small nuclear ribonucleoprotein) antibodies, positive antinuclear antibody (ANA) (1:1280, homogeneous pattern), and positive rheumatoid factor (16 IU/mL). Anti-cyclic citrullinated peptide (anti-CCP) antibodies, antineutrophil cytoplasmic antibody (ANCA), angiotensin-converting enzyme (ACE), and immunoglobulin G4 (IgG4) levels were negative or within normal limits (Table 1).

**Table 1:** Notable labs upon presentation to rheumatology ANA: Antinuclear antibody; SSA: Sjögren's-Syndrome-related Antigen A; SSB: Sjögren's-Syndrome-related Antigen B; ESR: Erythrocyte sedimentation rate; U1 SNRNP IgG: Small nuclear ribonucleoprotein immunoglobulin G

Pertinent Lab Data	Patient's Lab Values	Reference Range
ANA Ab Titer	1:1280	<1:40
SSA (U)	>8.0	<1.0
SSB (U)	>8.0	<1.0
Rheumatoid Factor (IU/mL)	16	<14
Centromere B Antibody	6.5	<1.0
ESR (mm/hr)	40	<=25
U1 SNRNP IgG (U)	21	0-19

**Figure 4:**
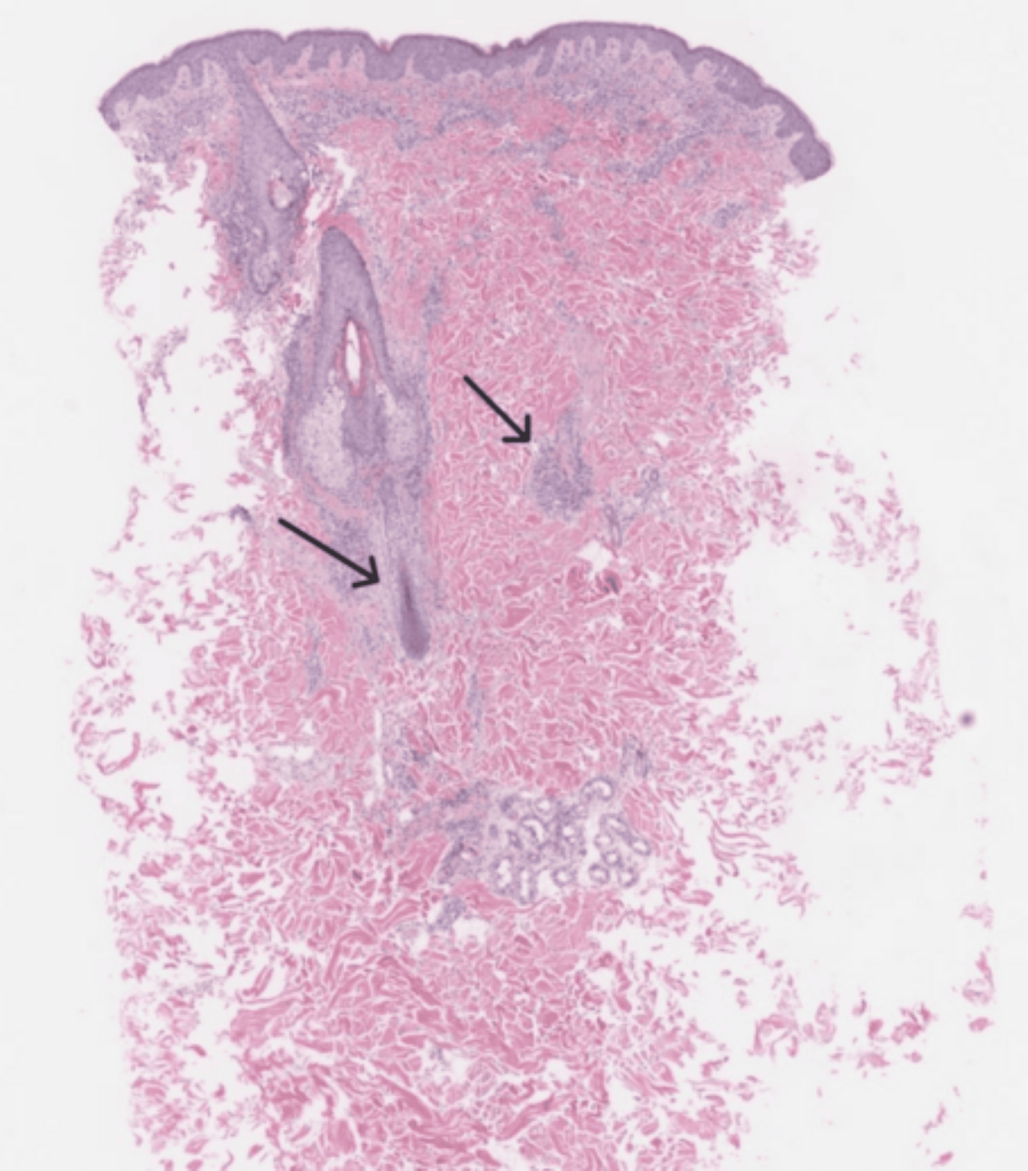
A 2x low-power hematoxylin & eosin staining - interface dermatitis, with arrows showing deep perivascular and periadnexal lymphoplasmacytic inflammation

**Figure 5:**
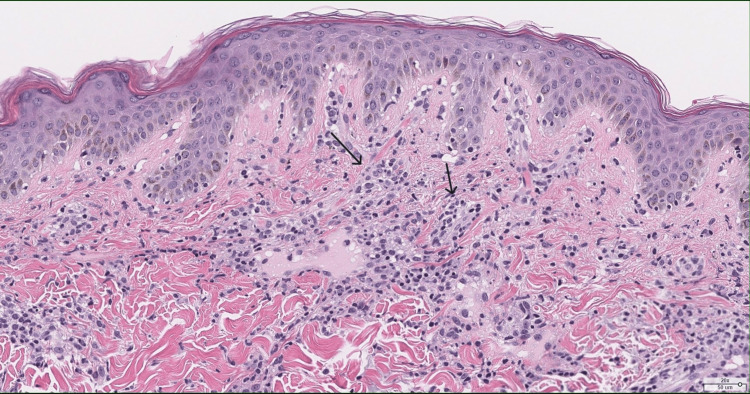
A 20x power hematoxylin & eosin staining - interface dermatitis, with arrows showing superficial perivascular and periadnexal lymphoplasmacytic inflammation

**Figure 6:**
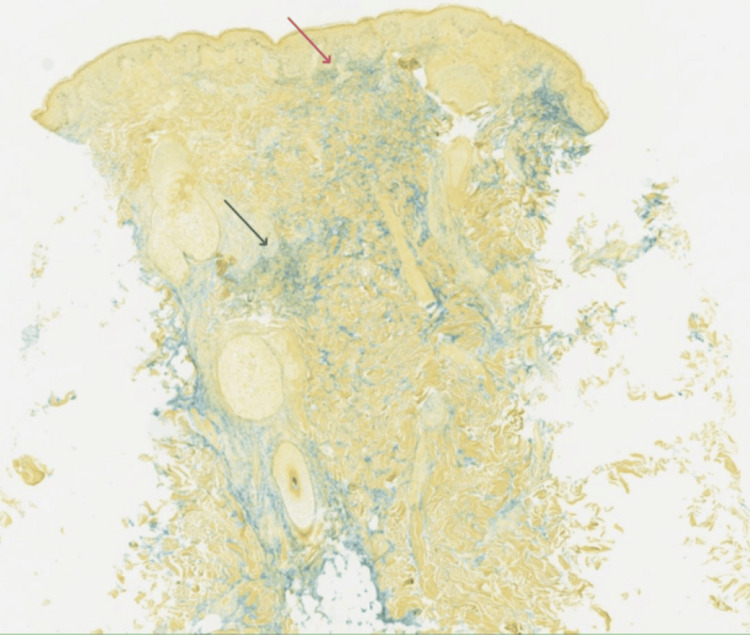
A 3x power colloidal iron special stain highlighting superficial (red arrow) and deep dermal (black arrow) mucin, alcian blue, and colloidal iron stain

A punch biopsy from a representative lesion on the right paraspinal back demonstrated interface dermatitis with superficial and deep dermal perivascular and perifollicular lymphoplasmacytic inflammation (Figures 4-5). Special stains (alcian blue and colloidal iron) revealed increased dermal mucin deposition (Figure 6). No evidence of malignancy was identified. The pathologist commented that the findings supported a diagnosis of connective tissue disease, most consistent with tumid lupus. 

Initial management included hydroxychloroquine (Plaquenil) 400 mg daily, methotrexate 15 mg weekly, prednisone 20 mg daily (tapered over eight weeks), topical tacrolimus 0.1% ointment twice daily, and intralesional triamcinolone (Kenalog) injections (5 mg/mL) for persistent lesions. Despite this multimodal approach for two months, the patient continued to develop new lesions, with only partial improvement in existing lesions. 

Given the inadequate response to conventional therapy, belimumab was initiated at a dose of 10 mg/kg intravenously every two weeks for the first three doses, followed by monthly infusions. Significant improvement was observed after the third infusion, with resolution of fever, marked reduction in the number and intensity of skin lesions, and decreased pain. After six months of belimumab therapy, the patient maintained substantial clinical improvement, with only occasional mild flares associated with cold exposure. 

## Discussion

This case presents several interesting diagnostic and therapeutic considerations. The clinical presentation of chilblain-like lesions in a patient with established Sjögren's syndrome, together with histopathological features of tumid lupus, illustrates the complex overlap that can occur within the spectrum of autoimmune rheumatic diseases [6,10]. 

The distribution of lesions in our patient is noteworthy, with involvement of both acral sites (characteristic of chilblains) and photo-distributed areas on the face and trunk (typical of tumid lupus). This mixed distribution pattern further supports the concept of an overlap syndrome, with features of both conditions [4,8].

The coexistence of multiple autoantibodies in our patient is also significant. While anti-SSA and anti-SSB antibodies are characteristic of Sjögren's syndrome, the presence of anti-centromere antibodies (typically associated with limited systemic sclerosis) and anti-U1RNP (anti-U1 ribonucleoprotein) antibodies (associated with mixed connective tissue disease) suggests an autoimmune overlap syndrome. This serological profile has been reported to correlate with a higher risk of extra-glandular manifestations in Sjögren's syndrome patients, including cutaneous involvement [10-12]. 

Distinguishing chilblains from tumid lupus can be challenging, as both conditions may demonstrate lymphocytic infiltration and dermal mucin deposition. However, the prominent interface dermatitis and significant mucin accumulation in our patient’s biopsy are more characteristic of tumid lupus [8,13]. The literature suggests that chilblains associated with autoimmune conditions often show histological features that overlap with lupus erythematosus, supporting the concept of a spectrum rather than distinct entities [7,8]. 

The therapeutic approach to chilblains in the setting of autoimmune disease typically includes conservative measures, calcium channel blockers, antimalarials, and immunosuppressants. Tumid lupus generally responds well to antimalarials and photoprotection [8,9]. However, the patient demonstrated only a partial response to conventional therapy, necessitating escalation to biologic therapy. 

Belimumab, a monoclonal antibody targeting B-lymphocyte stimulator (BLyS), is FDA-approved for systemic lupus erythematosus and has shown efficacy in Sjögren's syndrome in small studies [14,15]. The significant clinical improvement observed with belimumab in our patient suggests that B-cell-targeted therapy may be particularly effective in managing cutaneous manifestations at the intersection of Sjögren's syndrome and lupus. Recent literature supports the efficacy of belimumab in various cutaneous lupus subtypes, though data specifically for tumid lupus or chilblains in Sjögren's syndrome remain limited [15]. 

This case adds to the growing body of evidence suggesting that targeting the B-cell pathway may be beneficial in autoimmune overlap syndromes with prominent cutaneous manifestations. It also highlights the importance of recognizing the potential overlap between chilblains and tumid lupus in patients with underlying autoimmune disorders. 

## Conclusions

This case explores a rare instance of chilblain-like lesions with histopathological features of tumid lupus in a young woman with Sjögren's syndrome and serological evidence of autoimmune overlap. It illustrates the diagnostic challenges in characterizing cutaneous manifestations in autoimmune disorders and demonstrates the potential efficacy of belimumab in managing refractory skin lesions. The favorable response to B-cell-targeted therapy suggests that dysregulated B-cell pathways play a significant role in the pathogenesis of these overlapping conditions. Further research is warranted to better understand the relationship between Sjögren's syndrome, chilblains, and tumid lupus, and to establish optimal therapeutic strategies for patients with similar presentations. 
